# High-Consistency Foam Pilot Process for Producing Fibrous Cushion Material

**DOI:** 10.1021/acsengineeringau.5c00120

**Published:** 2026-04-28

**Authors:** Elina Pääkkönen, Tuomas Turpeinen, Olli-Ville Laukkanen, Baranivignesh Prakash, Jouni Paltakari, Jukka A. Ketoja

**Affiliations:** † 3259 VTT Technical Research Centre of Finland Ltd, Sustainable Products and Materials, P.O. Box 1603, Jyväskylä FI-40101, Finland; ‡ Aalto University, Department of Bioproducts and Biosystems, P.O. Box 11000, Aalto, Espoo FI-00076, Finland

**Keywords:** foam forming, piloting, cellulose fiber, rheology, cross-linking, microstructure, cushioning

## Abstract

High-consistency (HC) suspensions containing several weight% of fibers have been studied in the past but mainly in relation to water-laid web-forming technology. The severe flocking problems deteriorating web quality can be partly solved by using aqueous foam as a transfer medium instead of water. The processability of highly viscous HC foam and its potential to produce low-density fiber products are revealed only in large-scale dynamic conditions with the proper flow properties of the foam. For this reason, a pilot-scale study was conducted at 10–14% fiber consistency and a 3 m/min web speed using softwood, hardwood, and a 50/50 fiber mixture as raw materials. To better understand the effects of additives on foam processability and the strength of the final product, the following additives were selected: guar gum (GG), carboxymethyl cellulose (CMC), citric acid, and a combination of the latter two. These studies were supported by rheological measurements and X-ray microtomography analysis of the product structures. In rheological measurements, the HC fiber foams turned out to have significant elasticity with a high storage modulus. Interestingly, the highest consistency in the pilot studies led to the smallest mean floc size and total floc volume in the corresponding samples. The best strength properties were gained with citric acid and CMC. Overall, the findings indicate that HC foam demonstrates significant technological potential for producing sustainable fiber products with efficient water usage.

## Graphical abstract

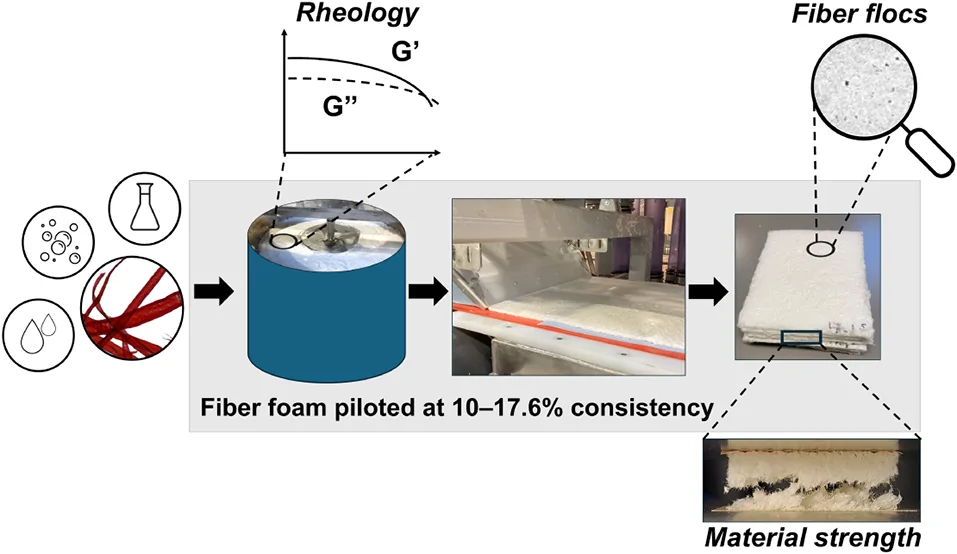

## Introduction

The green transition encourages us to promote new technologies that reduce our reliance on fossil fuels. One such effort is to find alternatives to expanded polystyrene (EPS) used in cushioning, which poses recycling challenges and is poorly biodegradable when it ends up in nature. Various initiatives are underway to replace plastics with easily recyclable, biodegradable, and sustainable fiber-based materials.^1–4^ Traditionally, fiber-based materials are processed using significant amounts of water, with paper production occurring at 0.5–2% consistency (percentage of oven-dry mass in suspension). However, to conserve fresh water due to global water scarcity, minimizing water usage is critical. Increasing fiber consistency has been a long-term goal in papermaking.^5–8^ However, the forming of high-consistency (HC) fiber suspension has been challenging due to the high viscosity and fiber flocking effects. Fiber suspensions are commonly described as having low, medium, or high consistency, but these categories do not have precise boundaries, and the terminology can differ depending on the context. In pulp processing, medium- (6–18%) or high-consistency (>18%) technology is currently used.^9^ In the case of web forming, Karvinen^5^ has called HC to be ca. 3% and ultrahigh, where consistency is 5–10%. Probably, the highest commercially used forming consistency is around 3% in the drying of chemical pulp.^10^

One interesting alternative is to utilize shear-thinning aqueous foam to improve processability at high consistency. In the case of fiber foams, we define HC as being ≥10%. HC foams have the potential to reduce water and energy consumption in current forming processes and to decrease equipment size,^11^ e.g., by shortening the vacuum and pressing sections. This innovation could be the key to a major advancement in the field. Foam forming is a water-efficient processing method that not only enables the production of lightweight materials from cellulose-based fibers but also expands the base of potential raw materials.^12,13^ In foam forming, wood fibers, water, and surface-active agents are mechanically mixed as aqueous foam with an air content of 40–80%. Then, the wet foam is pumped onto a wire or poured into a mold, and free water is removed; the remaining water is evaporated. Depending on the used fiber consistency in foaming and applied vacuum levels, the dewatering phase can lead to highly layered structures, the final structure having a denser, “skin-like” layer against the wire.^14,15^ Such intensive surface densification can be avoided in HC foam forming by keeping vacuum levels low and by starting drying right after forming. Moreover, the inner structure is also expected to become more even as the foam keeps fibers at least partly separated until drainage,^12,16,17^ and fiber flocs disintegrate under the shear forces imposed by the bubbles.^18^ When producing low-density structures, additional advantages of using HC foams are to retain the even structure without major shrinkage in the dewatering section^15,17^ and to avoid the collapse or expansion of the structure, especially in microwave drying.^19^

Resource-efficient processing is the pathway to manufacturing sustainable fiber-based products. Even though there has been active research around fiber foams, most of the studies have been performed on a laboratory scale. Translating these studies to the industrial scale is not straightforward without bridging the knowledge gap on a pilot scale. Moreover, dynamic pilot trials provide advantages, such as achieving a uniform distribution of highly viscous HC fiber foam. Similar sample quality would be challenging to attain in laboratory testing. The reported pilot studies have operated mainly at relatively low consistency (0.5–2%).^20–23^ Kiiskinen et al.^24^ and Torvinen et al.^25^ note that after wet pressing, the web solids content is typically 40–50%, and a further 2–3%-unit increase achieved by switching from water-laid to foam forming corresponds to an 8–12% reduction in the drying energy demand.

In this work, we show that high forming consistencies can be more easily achieved by using foam as a transfer medium. Compared to traditional HC water forming, foam forming can enable remarkably higher consistency levels with better end-quality and thus a broader product range. Our study was carried out at the pilot scale to ensure a proper rheology environment for HC foam. Producing equivalent samples at the laboratory scale was not feasible due to challenges in distributing HC foam in the sheet molds. This work consisted of two parts: (i) the effect of HC foam’s viscoelastic rheology on pilot workability and (ii) low-density product performance. Based on the identified gap in HC foam forming, the research questions are 1) How does high forming consistency affect fiber distribution uniformity and mechanical properties of lightweight fiber materials? and 2) How can HC foam processability and interfiber bonding be improved with selected additives, individually or in combination? We hypothesize that increasing consistency changes foam rheology so that more energy is needed to make the foam flow stable. On the other hand, consistency is expected to affect the homogeneity and mechanical properties of the materials. The lightweight fiber webs were produced using VTT’s SAMPO pilot machine with softwood and hardwood fibers at consistencies varying between 10 and 14%. From the oven-dried structures, we analyzed several structural and mechanical properties that are relevant for the cushioning material. To increase the final strength, carboxymethyl cellulose (CMC) together with citric acid (CA) was used. Additionally, to understand the role of HC foam rheology better, part of the studied fiber foams was reproduced in a laboratory for rheological characterization.

## Experimental Section

### Materials

Bleached softwood kraft (SW, pine, average fiber length 1.99 mm) pulp was obtained from Metsä Fiber, Äänekoski mill, Finland, and bleached hardwood (HW, eucalyptus, average fiber length 0.76 mm) pulp was received from Suzano, Aracruz mill, Brazil. Both pulps were first disintegrated at 4% consistency and then screw-pressed to increase the solids content of the pulp to ca. 30%. For most of the studies, SW and HW were used as a 50/50 mixture. After the screw-pressing, the pulps were full of flocs (see Figure S1 in Supporting Information). Fiber properties of the disintegrated pulps were analyzed with the L&W FiberTester Code 912 Plus (ABB, Lorentzen & Wettre, Kista, Sweden) and are reported in Table S1.

As a surfactant, a nonionic Simulsol SL10, an alkylpolyglycoside (MW 320 g/mol, purity 51%, Seppic S. A., France) was used. An aqueous surfactant solution of 10 wt % concentration was first prepared from the commercial formulation. This solution was used to add selected surfactant amounts to the total suspension volume in both the laboratory and pilot studies. The applied dosages, 4 g/L (TP1-TP7), 5.1 g/L (TP9-TP11), or 6 g/L (TP8), varied so that a similar target air content of the foam was obtained for all trial points. Moreover, guar gum (GG), carboxymethyl cellulose (CMC), or citric acid (CA) were used as additives to improve dry or wet strength and to help the processing of high-consistency foam. The detailed properties of the additives are described in Table S2. All additives were added at the end of the foaming operation. When using CA and CMC together (TP11), CA was added first and then CMC. The samples containing CA were cured in a 190 °C oven for 10 min to activate CA cross-linking, thereby fixing the binder in place and developing the final mechanical integrity of the structure.

The studied recipes, their compositions, and target fiber consistencies in pilot trials are listed in Table 1.

**Table 1:** Studied Recipes, Their Raw Materials, and Target Fiber Consistencies in the Pilot Trials and Lab Studies

Trial point	Lab studies	Studied furnish property	Pulp type	Target fiber consistency, %
TP1	x	Fiber type	SW	10
TP2	x		HW	10
TP3	x		50/50 SW/HW	10
TP4	x	Fiber type with GG 0.5% additive	SW	10
TP5	x		HW	10
TP6	x		50/50 SW/HW	10
TP7		Higher consistency with GG 0.5% additive	50/50 SW/HW	12
TP8			50/50 SW/HW	14
TP9		CMC 0.5% additive	50/50 SW/HW	10
TP10		CA 1% additive	50/50 SW/HW	10
TP11	x	CMC 0.5% + CA 1% additives	50/50 SW/HW	10

### Pilot-Scale High-Consistency Foam Process

The pilot-scale trials were conducted at the VTT SAMPO pilot facility in Jyväskylä, Finland. The pilot has been specifically designed for preparing nonpressed continuous webs.^24^ In the trials, the machine (Figure 1) consisted of the following unit operations: foam generation unit, pump, horizontal headbox, wire section, four vacuum boxes, two dryers, and sampling section. A drying fabric was used in one wire loop, covering both the forming and drying sections to avoid a gap between them and to prevent the wet fiber web from moving from one fabric to another. To maintain workplace safety while collecting wet pilot samples, it was necessary to halt the machine operation. Consequently, vacuum boxes or online dryers were not utilized in this study.

**Figure 1:**
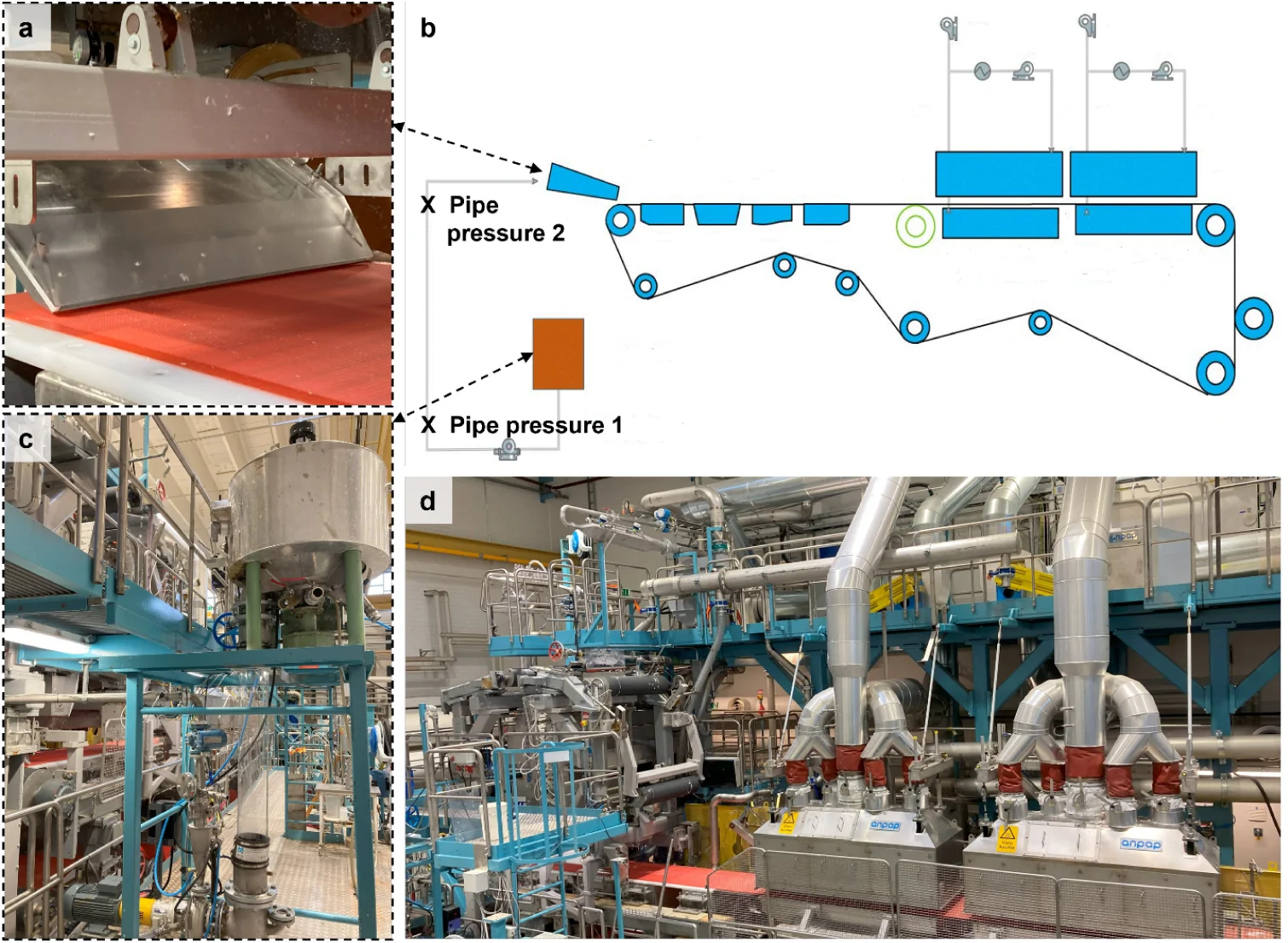
Pilot process in the HC foam trials: (a) Horizontal headbox with 8 mm opening, (b) pilot process layout marked with two pipe pressure measurement points, (c) foam generation unit, and (d) side view of the SAMPO pilot line.

HC foam was generated in a 200 L tank equipped with 0.33-m diameter down-pumping pitch blade turbines. The foaming tank was placed higher than the pump and was followed by a drop-leg section to generate the necessary suction pressure for pumping the high-consistency foam. A centrifugal pump was employed to transfer the foam to the headbox. From the pump to the headbox, a 3-m pipe with a 57 mm inner diameter was installed and equipped with pressure transmitters (Cerabar PMP21, in the 6-bar range, Endress+Hauser Oy), comparable to those in ref 26, but without the differential pressure transmitter. Pressure transmitters were placed at a 2 m distance in a horizontal pipe. The headbox width was 640 mm, and the slice opening was set to 8 mm. The target fiber consistency varied from 10% to 14% before foaming, and the obtained foam density was in the range of 150–220 kg/m^3^ that corresponded to an air content of 78–85% (Table S3). Foam density was controlled by the amount of surfactant and the mixing time. The pilot machine was operated in a Fourdrinier-former mode at a speed of 3 m/min.

Collecting material samples was carried out as follows: 1) A piece of forming fabric was placed on the drying fabric before the headbox, 2) a thick foam web was formed from the headbox on the forming fabric, 3) the pilot machine was stopped, 4) five parallel foam samples (ca. 640 × 500 mm) for each trial point were collected from the wire, and 5) the wet samples were dried in an oven at 80 °C.

### Analytical Methods

#### Foam Generation in the Laboratory

To measure the rheological properties of high-consistency foam, small batches of aqueous foams were generated in a laboratory-scale mixer (Netzsch, Hedensted, Denmark). The lab-scale testing facility comprised a transparent acrylic tank (0.43 m in height and 0.16 m in diameter), equipped with a top-entry impeller with three down-pumping pitched blade turbines.^11,27^ The initial volume was 1 L, including the screw-pressed pulp(s) and the same dosages of surfactant and additives as used in the pilot trials. The foaming was carried out at a rotational speed of 700–900 rpm until the targeted air content was reached.

#### Foam Rheology Measurements

From the generated foam, a small sample was taken to measure the rheological properties. A stress-controlled TA Instruments DHR-2 rheometer was used, equipped with a wide-gap vane-in-cup geometry. The four-bladed vane geometry had a diameter of 15 mm, while the cup had a diameter of 30 mm. The cup was 3D-printed to have a grooved surface to eliminate wall slip.^28^ The temperature of the samples (*T* = 25 °C) was controlled with a Peltier jacket connected to a Julabo AWC100 recirculating cooler.

Three types of rheological measurements were conducted: frequency sweep, amplitude sweep, and shear rate sweep measurements. Frequency sweep measurements were performed in the angular frequency range of 0.40 to 100 rad/s at a strain amplitude of 0.03%, ensuring that the measurements were performed in the linear viscoelastic range. In the amplitude sweep measurements, the strain amplitude was varied from 0.01 to 100% while keeping the angular frequency constant at 10 rad/s. Shear rate sweep measurements were performed both at increasing shear rate (from 0.1 to 100 s^–1^) and then at decreasing shear rate (from 100 to 0.01 s^–1^) as the flow behavior of the foam samples was expected to be dependent on the shear history of the sample, e.g., due to thixotropic effects.

#### Foam Air Content and Density

The air content of the foam was calculated from the foam density with the following equation: Air content (%) = (1 – ρ_foam_/ρ_liq._) × 100, where the liquid density ρ_liq._ ≈ 1000 kg/m^3^. From the laboratory-generated foams, a foam sample was taken with a cut-open syringe, and the final foam density was measured by weighing a 50.6 mL foam volume in the vane-in-cup. The air content of the foams varied from 77 to 82% when measured directly from the rheometer cup. In the pilot, the mixing in the tank was stopped prior to sampling when the foam level had reached the target, corresponding to ca. 80% air content. A foam sample was collected from the top of the tank with a calibrated container of known volume (3.56 L), and the foam density was determined gravimetrically by weighing. The air contents varied from 78% to 85% (Table S3), being the lowest for the trial points with consistency exceeding 10%.

#### Grammage, Thickness, and Density

The samples were conditioned overnight under laboratory conditions of 23 ± 1 °C and a relative humidity of 50 ± 2%. For the testing, the large pilot trial samples were cut into smaller, ca. 150 × 150 mm, samples with a bandsaw (Makita LB1200F). From each trial point, ten parallel samples were weighed, and their thickness was measured with a laser. The results are presented in Table S3. Due to variations in the sample thickness, the density was measured from the actual compression and z-strength test samples and is reported together with mechanical properties.

#### X-ray Tomography Imaging and Analyzes

The samples were imaged with an X-ray microtomography scanner (RX Solutions, Chavanod, France) to analyze the internal structure, including average pore size distribution and possible flocs. A 40 kV X-ray tube voltage and 16 W electron beam power were applied during the imaging. Each image had a pixel size of 5.8 μm, and 1440 projection images were captured over a full 360° rotation for each sample. The size of the imaged region was 5 × 5 mm. A representative region based on the visual evaluation was chosen. For the pore size distribution, we used the local thickness transform (LTT)^29^ to fit spheres into the void space, and the volume-normalized histogram of the LTT result defines the pore diameter distribution. In the XμCT data, flocs do not appear as uniform dark areas in the image and are therefore difficult to determine. A new method was developed to determine the mean floc size and the total relative floc volume from an XμCT image. Here, floc was defined as a region where the fibers were closer to each other than, on average, in the sample. As the obtained floc size varies depending on how big a difference in the interfiber distance is required compared to the average distance, the final size is obtained by systematic averaging over a range of tested threshold distances. The new algorithm is described in detail in Supporting Information.

#### Fourier Transform Infrared (FTIR) Spectrometry

To study the chemical structure of the produced pilot samples, the samples were analyzed with attenuated total reflectance Fourier transform infrared spectroscopy (ATR-FTIR, Nicolet iS50, Thermo Fisher Scientific Inc., Waltham, MA, USA) fitted with a single-reflectance diamond ATR crystal. ATR-FTIR is a nondestructive method and was performed directly on solid foam-formed samples using a semiquantitative approach by pressing the samples against a diamond crystal using a fixing screw. The solid citric acid monohydrate reference indicated peaks at 1743 cm^–1^ and 1693 cm^–1^. The measurements were mainly done from the top of the sample, and for the most interesting sample TP11, also the middle and bottom layers were analyzed. The main interest was to see the effects of CA and CMC additives on the samples.

#### Scanning Electron Microscopy (SEM)

The fiber-additive interactions as visible in the network structure of the samples were investigated by using field-emission scanning electron microscopy (Zeiss Merlin Gemini Field Emission SEM, Carl Zeiss Microscope, Oberkochen, Germany) operating at an acceleration voltage of 2 kV. A Leica EM ACE200 sputter coater was used for the preparation of the samples by sputtering them with a 5 nm layer of gold and palladium (Au + Pd). During imaging, an SE2 detector and a current of 60 pA were used. SEM images were taken from the top of the samples.

#### Z-Strength

Z-strength was measured using the Lloyd R10K universal tester (Lloyd Instruments Ltd., Bognor Regis, West Sussex, UK) from the samples cut to ca. 25 × 50 mm in size. The exact area was measured for each sample. Five parallel measurements were carried out for all trial points. Double-sided tape (Scotch 3M) was used to attach the samples to the probes. To ensure good adhesion between probes, tape, and the sample, slight precompression with a 10 N force was applied for 20 s for all other recipes except TP11, which required a longer 30-s precompression with a 20 N force. After this compression, the probes were pulled apart at a speed of 80 mm/min, and the average maximum force was measured. The z-strength (Pa) is the average peak force (N) divided by the sample area (m^2^).

#### Quasi-Static Compression Stress and Recovery

Compression stress and recovery were measured with a Lloyd R10K universal tester (Lloyd Instruments Ltd., Bognor Regis, West Sussex, UK). The employed sample dimensions were 75 mm × 60 mm (45 cm^2^), and four parallel samples were assessed. In the quasi-static pressing, the sample was first pressed to 10% of the initial thickness (at a speed of 0.1 × thickness/min), pressure was released for a recovery measurement, and then pressing was continued to 50% compression (at a speed of 1 × thickness/min). In both intermediate and final recovery measurements, the thickness of the sample was determined under a pressure of 250 Pa after the sample had recovered from compression for 1 min. The averaged strain–stress curves were plotted until 50% compression, and the energy absorption was calculated from the curves for each trial point as described in ref 4.

#### Final Characterization Plan

Different measurements were carried out from various trial points based on interest and are summarized in Table S4. The quasi-static compression stress and recovery, as well as tomography images were employed for all of the trial points. In z-strength measurements, the main interest was to compare the fiber types, higher consistency, and the effects of additives. In absorption measurements and FTIR, the main interest was in the trial points with additives. Rheology measurements were carried out later to understand the effect of fiber type, guar gum (GG), and the combination of CA + CMC on high-consistency foam rheology.

## Results and Discussion

In this section, we will first examine the rheological properties of HC foams to gain a better understanding of their behavior under piloting conditions. Subsequently, we summarize the main findings regarding the processability of HC fiber foams during pilot trials. Finally, we analyze the formed structures in detail, discuss the processing parameters that influence them, and summarize the results pertaining to z-strength, compression stress, and recovery.

### Rheological Properties of High-Consistency Foam

In the past, fiber foam rheology has been investigated mainly for dilute systems, up to 5% consistency.^30^ Low-consistency fiber foams are reported to be shear-thinning power-law fluids, whose flow behavior depends strongly on fiber type, consistency, and air content.^30–32^ Below, we provide new insights into the rheological behavior of HC fiber foams that exhibit viscoelastic behavior and whose rheological behavior cannot thus be described by viscosity alone.

In our study, some of the foam recipes that were produced on a pilot scale were also prepared on a laboratory scale to examine their rheological properties. All the foams with air content of 77–82% and consistencies of 10% presented highly elastic behavior (*G*′ ≫ *G*″), as shown in Figure 2a and 2b. The highest stiffness over the entire measured frequency range (Figure 2b) was seen with the sample including long fibers, Recipe 1 (SW) and Recipe 4 (SW + GG), and the lowest stiffness for Recipe 2 (HW) and Recipe 5 (HW + GG) due to the short fibers, with guar gum having more effect on short fibers. In both amplitude and frequency sweeps, SW/HW mixtures with GG (Recipe 6) and CA + CMC (Recipe 11) were almost identical when compared with each other, and also a bit higher than (Recipe 3) SW/HW without any additives. These results indicate that fiber length was more dominant for the fiber foam stiffness than additives or small variations in the air content, but with the shorter fibers, the effect of additives also became important. Otherwise, quite similar frequency dependences of *G*′and *G*″ were seen with all the samples, and no significant differences were recorded. Interestingly, the amplitude sweep data (Figure 2a) show that the strain amplitude at which the loss modulus *G*″ becomes larger than the storage modulus *G*′ is almost equal for all of the tested foams, meaning that roughly the same amount of deformation needs to be applied to make these foams predominantly liquid-like and flowable.

**Figure 2:**
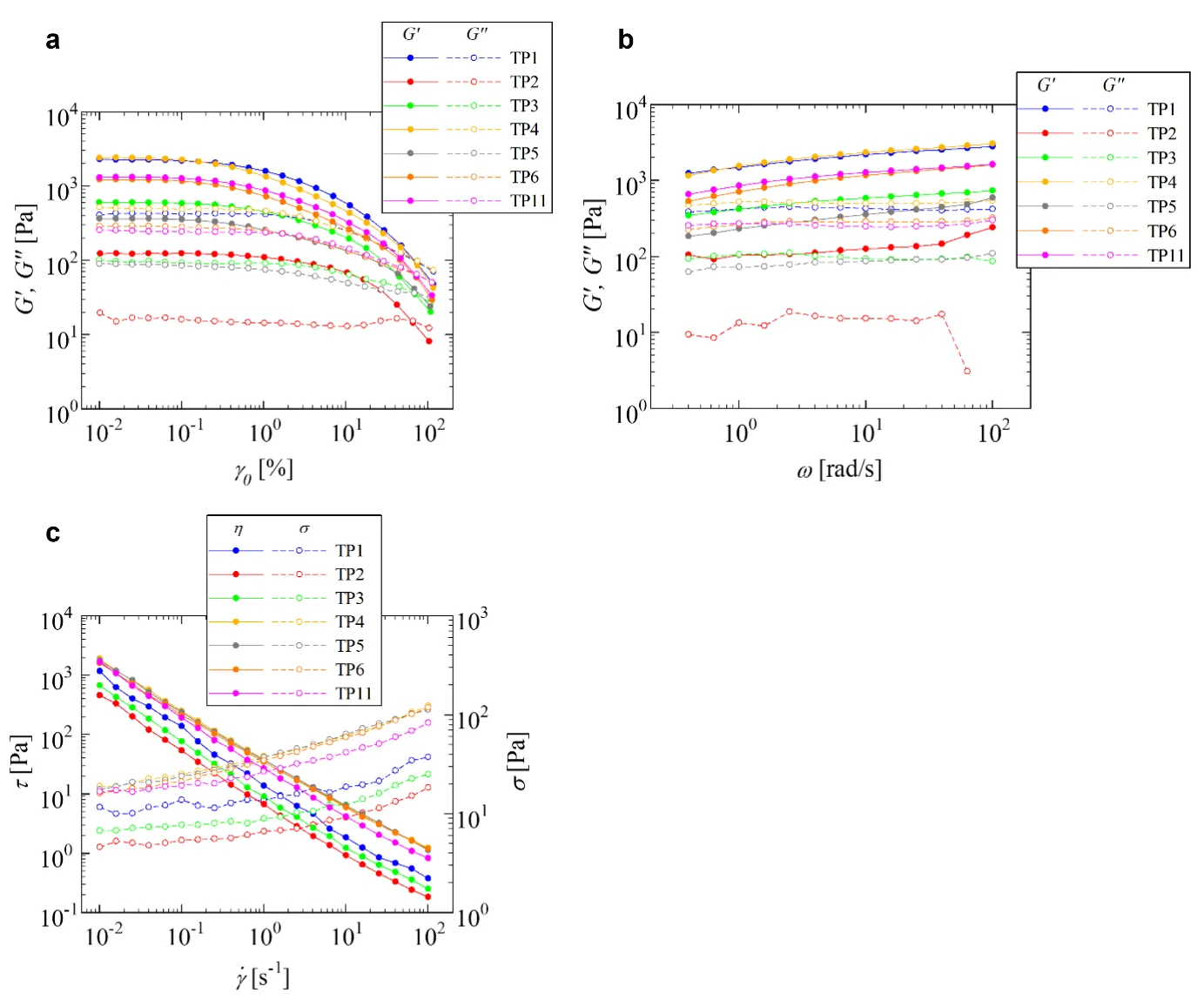
Rheological behavior of the foams produced in the laboratory corresponded to the air contents of TP1:80.6%, TP2:77.2%, TP3:77.4%, TP4:80.3%, TP5:78.3%, TP6:81.6%, and TP11:81.2%. (a) Amplitude sweep, (b) frequency sweep, and (c) shear rate sweep data. The test points included all fiber types, SW (Recipe 1), HW (Recipe 2), and SW/HW (Recipe 3) without GG and with GG (Recipes 4–6), as well as SW/HW with CMC and CA (Recipe 11).

All of the foams presented strong shear-thinning behavior with yield stress, as evidenced by the plateauing of the shear stress (σ) values at low shear rates (Figure 2c). Foams with GG (Recipes 4–6) had higher shear stress and shear viscosity (η) than foams without additives (Recipes 1–3). When foams with GG were compared with the Recipe 11 foam with CMC + CA, shear thinning was more pronounced in the latter one. The foams without additives had higher shear stress when longer fibers were used, and the shear-thinning behavior was similar to that of GG foams. It can be concluded that the tested fiber foams were highly elastic and strongly shear-thinning, and both additives and fiber properties affected the rheological behavior of the foams. At a consistency of ≤2%, it has been previously shown that the viscosity of fiber foam was about double that of plain aqueous foam, which is a significantly less increase than in plain aqueous fiber suspensions, where viscosity rises five times greater than that of water due to continuous fiber contact during shearing.^31^ The HC fiber foams studied here exhibited a high storage modulus and behaved more like solid materials than liquids. Nevertheless, even though the viscosities of the HC foams were high at low shear rates, they strongly decreased with increasing shear rate, improving their pumpability under the relevant process conditions.

According to our measurements, the rheological behavior of the HC foam can be more even than that of the HC water suspension, where the local viscosity varies tremendously depending on consistency and flow conditions, as summarized by Karvinen.^5^ In practice, sufficiently high shear rates need to be ensured to get the material moving out of the tank and into the pump. The high storage modulus can indicate that the fiber web is more stable, but on the other hand, it may cause difficulties in processing. Processing of the fiber foams may also affect the air content of the foam. To achieve all three—good foam stability, processability, and final strength—the SW/HW fiber mixtures are recommended at high consistencies.

### General Process Runnability and Control Parameters

The processing of HC foams presents new challenges for process equipment due to the elevated viscosity and air content. Previous studies^13,23,24,33^ have documented the piloting of foam forming at low consistency levels (<5%), achievable with minor changes to water-laid process units. These modifications include, e.g., installation of larger tanks for foam generation, the addition of gas-removal systems to pumps, enhancements in dewatering capacity, and the implementation of foam quality control systems. However, when focusing on the production of porous materials using high-consistency foams, some of these process changes may not be necessary.

Key control parameters at the pilot scale include fiber consistency in water, foam density, flow rate, and headbox opening. In our previous trials,^11^ foam density and machine speed were chosen as variables. In these pilot trials, the machine speed was kept constant at 3 m/min, and the target foam density for all trial points was 200 kg/m^3^, varying from 150–220 kg/m^3^ to achieve proper mixing. Processing involved weighing part of the screw-pressed fibers, adding water and surfactant, and mixing in the foaming tank; the rest of the fibers were added after premixing and foaming until the target volume was reached. With the current setup, the foaming times varied from 20–30 min, and in general, higher consistencies required longer mixing times. For most of the trial points, the surfactant dosage was 4–5.1 g/L, but for the highest consistency, the surfactant dosage had to be increased to 6 g/L to reach the target air content in a reasonable time. The foam quality during mixing was observed visually. SW, HW, and SW/HW mixtures were tested to compare the processability of fiber types at high consistency. The foam made with short HW fibers appeared smoother and less viscous than the foam with SW fibers or at consistencies above 10%, which appeared uneven and clumpy.

The pump speed was manually operated with a variable speed drive, and the achieved flow rate was monitored as an outcome. The target was to keep the foam flow as constant as possible, without any visible waves or empty areas, when the foam mat was coming out of the headbox. Flow rates varied from 0.20 to 0.39 L/s, corresponding to 0.35–1.03 L/s in the atmosphere, with pump rpm ranging from 1200 to 3950. With these settings, an even foam mat was gained at most of the trial points. At 10% consistency, all furnishes were quite easily processable with the selected pump and headbox opening. Figure 3 presents pump rotational speed as a function of fiber consistency and two different situations in which: 1) flow did not match the machine speed, and 2) flow was even. Pump rotational speed was found to be nearly linearly dependent on increasing consistency. The lowest pressure difference in the pipe was 3.2 kPa with TP5 (HW + GG), and the highest one was 11.1 kPa with TP8, having also the highest consistency. Also, the use of CMC and CA made the foam more challenging for pumping, probably due to the higher viscosity and slightly lower air content and thus deteriorating flow properties. The main challenges were related to some clogging inside the headbox or pump, causing streaks or empty spots in the formed web or poor flow rate. Additives like guar gum and CMC improved foam processability, making it smoother and easier to pump, and the foam flowed more evenly out of the headbox. The pilot trials confirmed that porous materials can be produced with HC foam with this kind of machinery, but more work would be needed to find optimal conditions and to verify foam stability for different processing steps.

**Figure 3:**
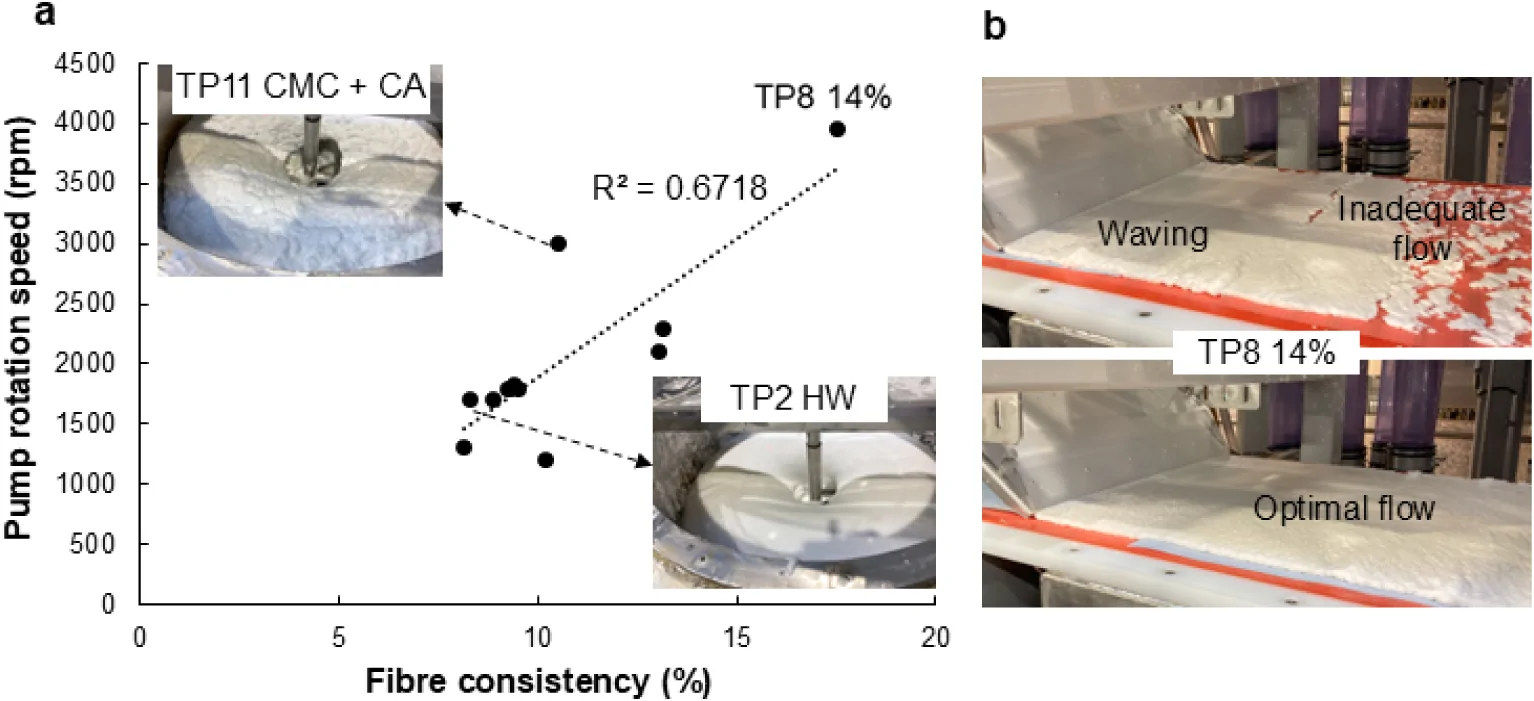
(a) Pump rotation speed as a function of the fiber consistency. TP2 foam was produced with HW, TP11 with SW/HW including CMC and CA, while TP8 SW/HW + GG was prepared at the highest consistency. Fiber consistency refers to the actual measured consistency. The pump rotational speed was manually adjusted to ensure even flow from the headbox, and the presented value corresponds to the optimal flow. (b) The upper image illustrates two undesirable scenarios: a high flow rate resulting in waving, and a low flow rate leading to empty areas. The lower image shows an optimal scenario where the flow rate matches the machine speed, resulting in an even flow. For TP8 SW/HW + GG, the target consistency was 14%. The red wire was the drying fabric, and a piece of blue wire was the forming fabric on which the samples were collected.

### Microstructure of the Formed Samples

A closer look at the dried pilot samples (Figure 4a) was obtained with SEM and reveals the effect of additives on the top structure (Figure 4b–f). The samples corresponding to TP6, TP9, and TP11 contain film-like structures, especially near the fiber joints, thus further strengthening the fiber network. The sample (TP11) with both CMC and CA appeared to have more bonding films (Figure 4f) than the sample containing only CMC (Figure 4d). At high magnification, dark spots could be seen on the fiber surfaces of the samples containing CA. The curing time might have been too long or the temperature too high, causing darkening and turning the sample surface yellow. Similar, but not so pronounced, film-like structures were also seen in our previous study,^4^ originating from poly­(vinyl alcohol) or fine particles from CTMP.

**Figure 4:**
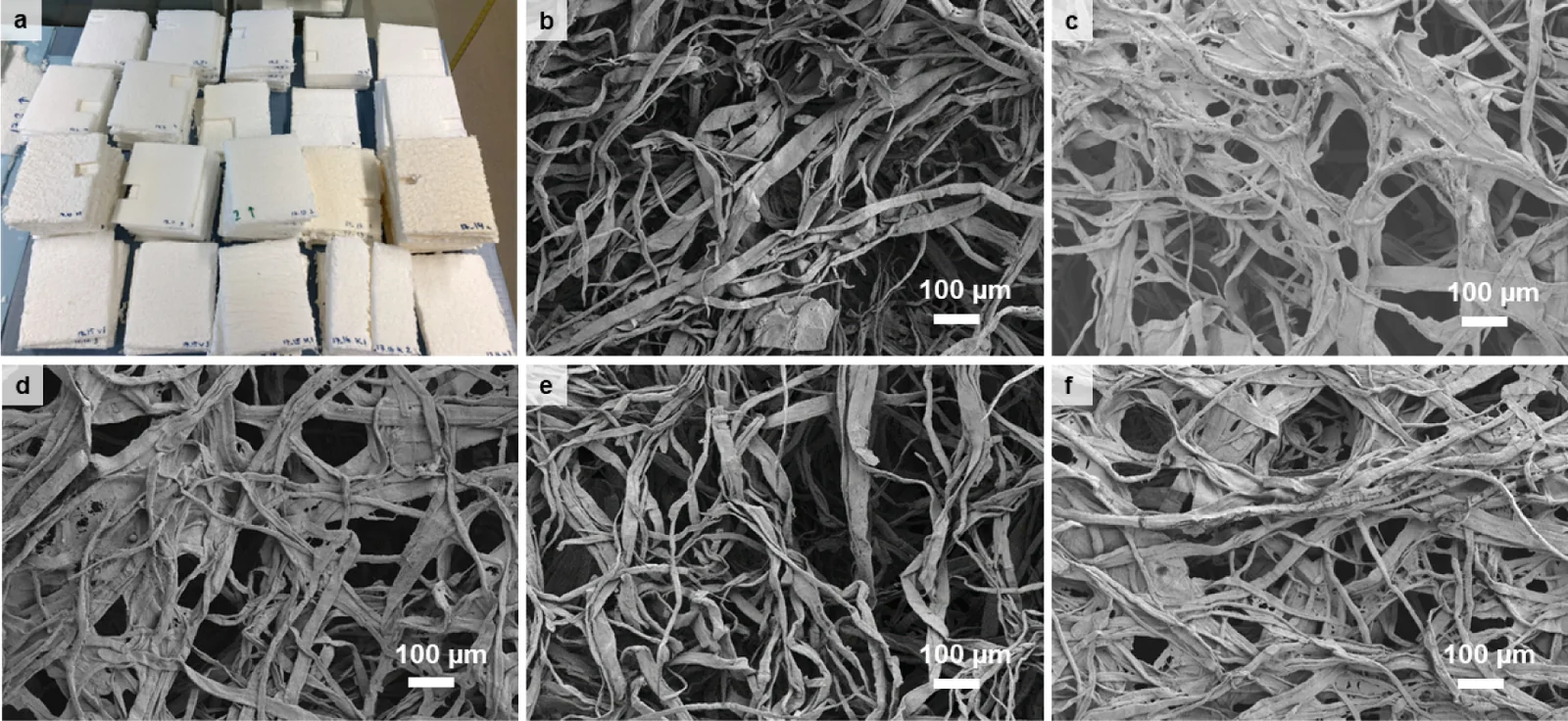
(a) Small 150 × 120 mm samples were cut from large pilot samples for further analysis. SEM images were taken from the top of the samples made from the SW/HW mixture as follows: (b) TP3 without additives, (c) TP6 with GG, (d) TP9 with CMC, (e) TP10 with CA, and (f) TP11 with CMC + CA.

Despite the high forming consistency, the structures had a low average density (Table S3) due to the low foam density (i.e., high air content in the foam). However, careful tuning of the mixing conditions was required for material homogeneity. Suitable conditions depended not only on the consistency but also on the fiber type, their preprocessing, mixing setup, and used additives. When the conditions were not optimal, visible fiber flocs or knots appeared in the dried samples. Before foaming, the pulp was screw-pressed to ca. 30% dry solids content, which gave rise to dense flocs that probably did not fully open during the subsequent processing steps. In other words, the mixing and forming operations were not considered as primary sources of the flocs. From the processing point of view, the largest fiber floc diameters of 6–9 mm blocked the local headbox flow and caused visible traces in the formed web. These acted as weak points in the dried samples. Another possible source of flow disturbance was seen at a low flow rate when the pump was operated outside its optimal range. Therefore, avoiding fiber flocs is essential to ensure both smooth processing and an even end product.

Fiber flocs were seen in most of the samples. Figure 5a–c shows that flocs had different sizes depending on the fiber type. The sample made from SW (Figure 5a) had larger but fewer flocs than the sample made from HW (Figure 5b). When both pulps were mixed (Figure 5c), flocs with a wide size variation could be seen. To get a better picture, the tomography images (Figure S3) were utilized to determine regions with lower-than-average fiber–fiber distance, which allowed determining the average floc size and their total relative volume (for method details, see Supporting Information). However, the size of the analyzed area was only 5 × 5 mm, so the largest flocs could not be recorded reliably. The mean floc size varied from 200 to 630 μm (Figure 5e), being smallest at high consistency and largest with SW. When GG was used as a viscosity modifier, both the average size and the relative volume of the fiber flocs were reduced for SW (TP4) but not for HW (TP5). Fewer flocs were seen in the sample formed at high consistency (TP8, 17.6%, Table S3), probably due to the higher shear forces during processing. The lowest relative volume of the fiber flocs (<3%) was found in TP8 (14%) and TP11 (CMC + CA). The role of the fiber flocs needs to be accounted for when considering the strength results. In summary, the use of longer fibers, viscosity modifiers, and increased consistency can help to improve structural homogeneity.

**Figure 5:**
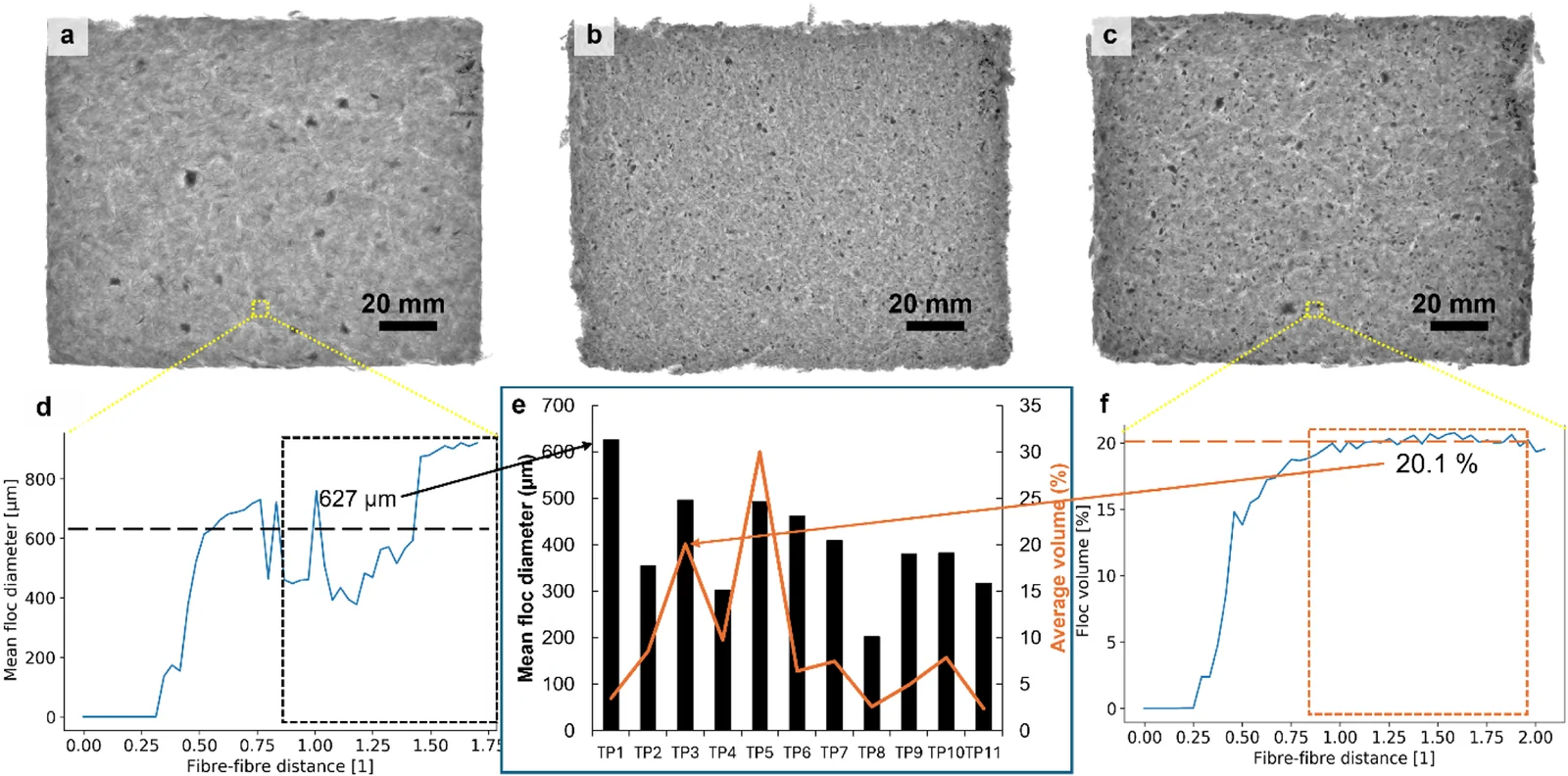
Backlight images of three samples on a light table visualized the differences in flocs within them: (a) TP1 SW sample had few but quite large flocs, (b) TP2 HW sample had a high number of small flocs, and (c) TP3 SW/HW sample contained flocs with varied sizes. A new algorithm was developed to analyze the flocs from the XμCT images. By plotting mean floc diameter (d) and relative total floc volume (f) against normalized fiber–fiber distance and averaging both over the shown distance interval, final results for all trial points (e) were obtained.

### Strengthening by Cross-Linking

Previous studies^34–38^ have shown that citric acid cross-links cellulose fibers and can be used, e.g., to enhance wet/dry strength and shape stability in water. Additionally, cross-linking fibers with CA and together with CMC has been reported earlier.^39,40^ Our research focused on the cross-linking potential of CA, CMC, and the combination of these for low-density structures under pilot conditions. Figure 6a illustrates that using CA (blue spectrum) produced a new FTIR peak at 1720 cm^–1^, and an increased peak at 1592 cm^–1^. These were even more prominent when CA was combined with CMC (green spectrum), potentially indicating new C=O bonds. However, FTIR analysis from the wire side revealed only minimal peaks (Figure S2), suggesting that additives remain mainly near the top surface. The underlying driving mechanisms are probably similar to those for binder migration during paper drying, which is a commonly known challenge in the paper industry, especially in coating operations.^41^ Here, the additives may migrate along the capillary-driven water flow toward the evaporating top surface, leading to their surface enrichment. However, the underlying mechanisms to understand additive migration would need further investigation. Overall, TP11 (green spectrum) with CA and CMC additives showed the strongest peaks, potentially indicating new ester and carboxylate bonds. However, the signal associated with ester groups may originate from both cross-linked and non-cross-linked citric acid. Thus, the cross-linking could not be concluded based on FTIR peaks alone but required further evidence from water absorption and shape-stability tests. These results (Figure S4) showed that the sample TP11, including CMC + CA, kept its shape the best and did not collapse during testing, which suggested the presence of CA cross-links with cellulose and CMC.

**Figure 6:**
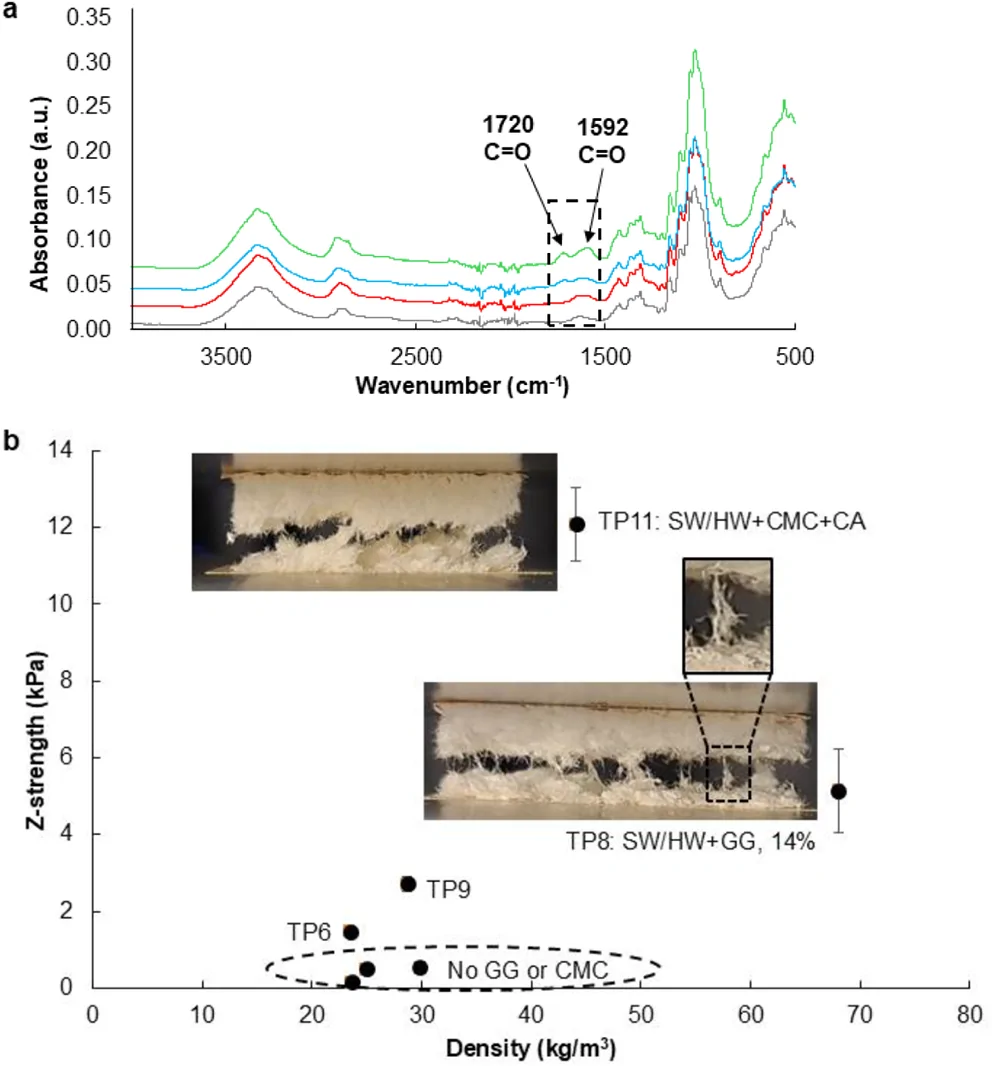
(a) FTIR spectrum obtained from the top of the foam-formed SW/HW samples, all foamed with Simulsol. The gray spectrum (TP3) stands for the reference sample without additives, the red spectrum (TP9) for the sample with CMC, and the blue spectrum (TP10) for the sample with CA. The green spectrum (TP11) with CMC and CA showed the most pronounced peaks at wavenumbers of 1720 cm^–1^ and 1592 cm^–1^. (b) Z-strength as a function of density for the selected trial points. Error bars show the standard deviation. TP1–3 prepared without GG or CMC presented very low strength, while the additives GG (TP6) and CMC (TP9) increased the strength somewhat. For TP8, some fiber roping was visible during the fracture process. TP11 with both additives had the highest z-strength.

The higher internal strength of the cross-linked structures is indicated by the measured z-strength in Figure 6b and Table S5. Without GG and CMC additives, the z-strength was rather low, being the lowest for TP2 (HW). The addition of GG increased the z-strength from 0.53 kPa (TP3 SW/HW) to 1.5 kPa (TP6), while with CMC, the strength improved to 2.7 kPa (TP9). In the case of TP8, the higher consistency led to higher density, also improving the relative bonded area and z-strength. When a closer look is taken at the photo of the sample, it seems that fibers have bundled into longer ropes within the structure. The most significant improvement was gained with the combination of CMC + CA (TP11), increasing the z-strength to 12 kPa, which indicates potential cross-linking in the structure. Previously, it has been shown^42^ that besides bond strength, the fiber alignment and internal microscale structure can affect the z-strength. Therefore, the reduced number of flocs in TP8 and TP11 samples may have contributed to their high z-strength. However, as the strength increased, the variability of the results also increased.

### Compression and Cushioning Behavior

The potential end-product quality and cushioning properties of the prepared materials were evaluated based on measured compression stress, energy absorption, and thickness recovery after compression. Compression stress is known to scale roughly by the square of density.^4,43^ In the pilot trial samples, there were large variations in the density, especially when consistency was increased. Table S3 lists the average value and standard deviation for each trial point, based on measurements from ten parallel samples. In Table 2, the measured single-sheet density is reported along with the corresponding strength, which may differ from the reported average value for all parallel samples. The basis weight and density of the pilot-scale samples varied from 128 g/m^2^ to 668 g/m^2^ and from 21.6 kg/m^3^ to 66.6 kg/m^3^, respectively, both being the lowest for TP2 (HW) and TP8 with the highest consistency. The thickness of the samples varied from 4.5 to 13.4 mm, and the production of thicker samples would be possible with a larger headbox opening. To compare the compression stress for different trial points, the measured values were normalized by the squared density (Table 2). From the normalized results illustrated in Figure S5, it can be concluded that SW gave higher stress at 50% compression than HW, and the results for the mixture of the pulps were in between. The addition of GG increased the maximum stress for all of these cases.

**Table 2:** Summary of the Final Product Properties Measured from a Single Pilot Sheet Sample for Each Trial Point

TP	Formulation	Density, kg/m^3^	Comp. stress at 50%, kPa	Normalized comp. stress,^b^ kPa	Recovery, %	*C* ^a^, -
TP1	SW	25	6.16	8.88	79.8	7.06
TP2	HW	24	3.91	6.11	69.7	5.58
TP3	SW/HW	26	5.93	7.90	71.9	4.91
TP4	SW + GG 0.5%	29	11.9	12.8	77.7	4.63
TP5	HW + GG 0.5%	26	7.25	9.65	71.0	6.47
TP6	SW/HW + GG 0.5%	22	5.54	10.3	73.4	5.21
TP7	SW/HW + GG 0.5%, 12%	38	17.8	11.1	77.2	4.87
TP8	SW/HW + GG 0.5%, 14%	43	31.3	15.2	74.5	4.83
TP9	SW/HW + CMC 0.5%	26	9.83	13.1	76.2	4.73
TP10	SW/HW + CA 1%	25	6.83	9.68	71.5	6.40
TP11	SW/HW + CMC + CA	41	39.3	21.0	77.0	4.05

a Cushion factor, *C*, is calculated for the 50% compression strain from the equation $C=\frac{\sigma_{m}}{E}$ , where σ_m_ is the maximum stress caused by the compression, and *E* is the absorbed energy per unit volume.

b The compression stress was normalized to a reference density of 30 kg/m^3^ according to σ_30_ = σ_measured_ *×* (30/ρ_actual_)^2^.

The effect of the forming consistency and different additives on compression stress is shown in Table 2. The higher forming consistency increased the compression stress together with the sample density. When comparing density-normalized results, higher compression stress was reached with higher consistency. This could be due to the more even stress distribution inside the structure since there were fewer flocs in those samples. The SW/HW sample with CA had a comparable compression stress to GG. With CMC, the compression stress was slightly higher. When both CA and CMC were used, a significant increase was achieved, probably because of successful cross-linking of all material components. Overall, the compression stress results are similar to or slightly higher than those for LC foam prepared at laboratory scale from SW in our earlier study.^4^

For packaging, cushioning performance depends not only on the material compression strength (σ) but also on how well the material absorbs the impact energy (*E*). This is often described by the cushion factor, *C* = σ/*E*, meaning that absorption is better when the cushion factor is low. Typically, for polymer foams like expanded polystyrene or polyethylene, the minimum cushion factor varies in the range of 2.5–4.^44,45^ For random fiber networks, the prediction for the theoretical minimum is 4.368.^4^ On the other hand, resilience refers to the ability of the material to recover after compression.

Table 2 presents the final material density, compression stress as such and after normalization, thickness recovery, and cushion factor. The lowest observed cushion factor was 4.05 for SW/HW with CMC + CA (TP11), and the highest was 7.06 for SW (TP1). This high value could be explained by the large pores inside the fiber network. In a previous study,^46^ the pores were larger in foam-formed papers than in water-formed samples, and their size correlated with the bubble size. On the other hand, the fiber type had a greater influence on pore size distribution than minor variations in bubble size distribution. For HC foams with a high storage modulus, a similar correlation between the bubble size and the mean pore size is not obvious. Moreover, it is difficult to measure the bubble size reliably at high consistency by using normal methods. For this reason, only the pore size distribution was analyzed for the selected samples. The samples TP1–TP3 made of different fiber types all had similar density, yet still there were differences in the pore size distribution, as presented in Figure 7. The SW sample had the largest proportion of large pores (>500 μm), whereas the pore size distribution for HW followed the SW distribution for small (<250 μm) and medium-sized (250–500 μm) pores but lacked the large ones. Curiously, the SW/HW mixture had fewer medium-sized pores than either of these two pulps. The pore size distribution became narrowest with the CMC and CA additives with the highest density. In Table 2, the measured cushion factors were in the same order: 7.06 (SW), 5.58 (HW), 4.91 (SW/HW), and 4.05 (SW/HW with CMC + CA). This reflected the effect of the large pores on the initial slope of the corresponding stress–strain curves, as shown in Figure S6a1. When considering all trial points, a weak linear correlation was observed between the cushion factor and pore diameter, with an *R* ^2^ value of 0.568. A higher correlation was not expected since energy absorption could also be affected by membranes, flocs, and fiber orientation, for example, besides the pore size distribution.

**Figure 7:**
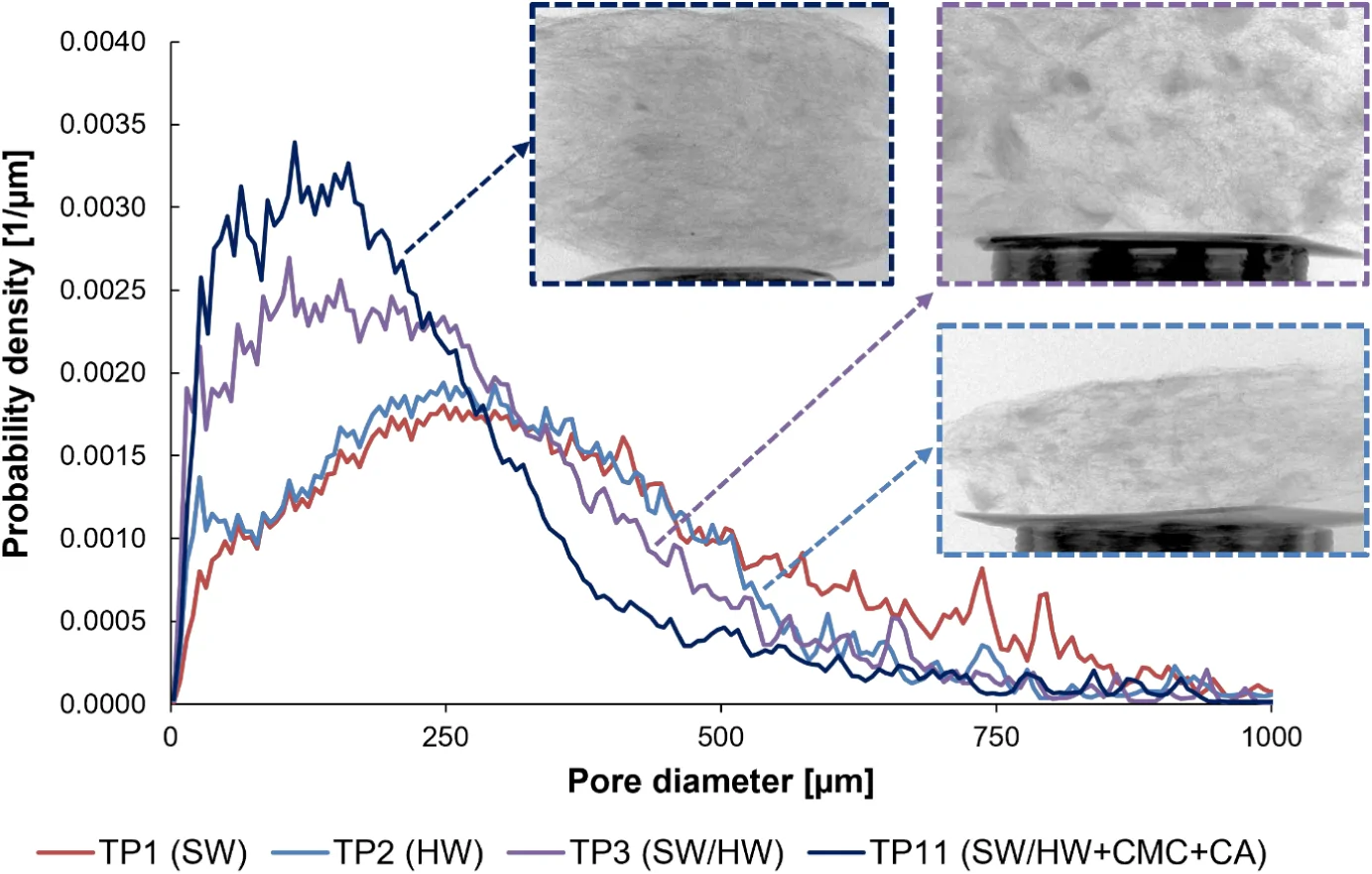
Pore size distribution of the selected trial points and X-ray tomography images visualized using Voreen software. Contrast of the tomography images was adjusted to visualize the flocs better. The densities of the samples were 25 kg/m^3^ (TP1), 24 kg/m^3^ (TP2), 26 kg/m^3^ (TP3), and 41 kg/m^3^ (TP11).

Besides the cushion factor, the pulp type also affects the thickness recovery after compression (Table 2). The highest recoveries were achieved with SW and SW/HW mixtures, especially with the additives. The lowest recovery was observed in the most brittle samples: HW, HW + GG, and SW/HW + CA. The sample with CA was probably not cross-linked well because of its rather low density and large pores. The further addition of CMC to the recipe almost doubled the density and provided a higher internal strength. Interestingly, SW/HW mixtures, especially those with GG, CMC, and CMC+CA, had the best energy absorption among the tested trial points. Overall, the best cushion factor value of 4.05 is comparable with expanded polyethylene^44^ and earlier best results with LC foams,^4^ thus indicating the potential of HC fiber foams for cushion applications.

## Conclusions

The primary motivation for enhancing consistency was to achieve a more water- and energy-efficient process, thereby reducing the size of the equipment and maintaining a uniform density distribution in prepared materials. Our dynamic pilot-scale trials provided an industrially realistic environment in which to study the processability of high-consistency (HC) foams. HC foam forming enabled the production of lightweight fiber materials with a significant reduction of freshwater usage compared with low-consistency methods. For example, the water amount needed to produce one ton of dry material is 99 m^3^/t at 1% consistency and only 6.1 m^3^/t at 14% consistency (Table S6), when neglecting process details such as water circulation that reduce the freshwater demand. Moreover, high solids content after forming is particularly important when aiming at low-density material without a density gradient because high vacuum or wet pressing is not suitable for water removal in such a case.

The pilot samples were formed successfully at 10–17.6% fiber consistency. The HC foams showed elastic rheological behavior, and an increase in consistency resulted in a higher demand for pumping energy. Interestingly, the samples produced at the maximum of the above consistency range showed both the smallest mean floc size and the lowest total floc volume, which indicated the significance of the flow shear forces for processability. The appearance of flocs also depended on the fiber type, additives, and other processing conditions. It is advisable to utilize SW/HW fiber mixtures in HC foam to ensure good foam stability, processability, and final product strength.

HC forming resulted in quite even fiber distribution and excellent mechanical properties, especially when using additives like carboxymethyl cellulose (CMC) and citric acid (CA). The additives also improved interfiber bonding, as shown by the increase in z-strength. The highest compression stress and lowest cushion factor, suggesting optimal impact energy absorption, were achieved when these additives were combined. The selection of additives affects not only processability and material strength but also energy efficiency. Even though the combination of CA and CMC gave the best results in this study, citric acid typically requires curing at an elevated temperature (>160 °C) to form ester cross-links with cellulose. A simultaneous use of catalysts may reduce the effective curing temperature to 120–140 °C, improving energy efficiency. Other additives could be also considered based on the end-of-life requirements.

In the produced porous materials, fiber flocs did not significantly affect the strength properties. However, if HC foam is intended for other applications, such as cardboard or thin paper products, the presence of fiber flocs should be avoided. This would require further optimization of the process equipment and testing of a larger variation of additives.

## Supplementary Material



## Notes

Detailed information about raw materials and additives, basic properties of pilot samples, final characterization plan, FTIR spectra, description of the method used for floc analysis from X-ray microtomography images, water absorption measurements, mechanical test results, and estimated water amount in a fiber suspension at varied consistency (PDF)
