# Supplementary material for: High-Consistency Foam Pilot Process for Producing Fibrous Cushion Material
Source: ACS Eng Au. 2026 Apr 28;6(4):519–30. doi: 10.1021/acsengineeringau.5c00120 (PMC13502054; doi:10.1021/acsengineeringau.5c00120)
Supplement: Supplementary file 1 [file eg5c00120_si_001.pdf]

SUPPORTING INFORMATION

High-consistency foam pilot process for producing fibrous cushion material

Elina Pääkkönen[a], Tuomas Turpeinen[a], Olli-Ville Laukkanen[a], Baranivignesh Prakash[a], Jouni Paltakari[b], Jukka A. Ketoja[a]

[a] VTT Technical Research Centre of Finland Ltd, Sustainable Products and Materials, P.O. Box 1603, FI-40101 Jyväskylä, Finland

[b] Aalto University, Department of Bioproducts and Biosystems, P.O. Box 11000, FI-00076 Aalto, Espoo, Finland

*Corresponding author: jukka.ketoja@vtt.fi

Raw materials

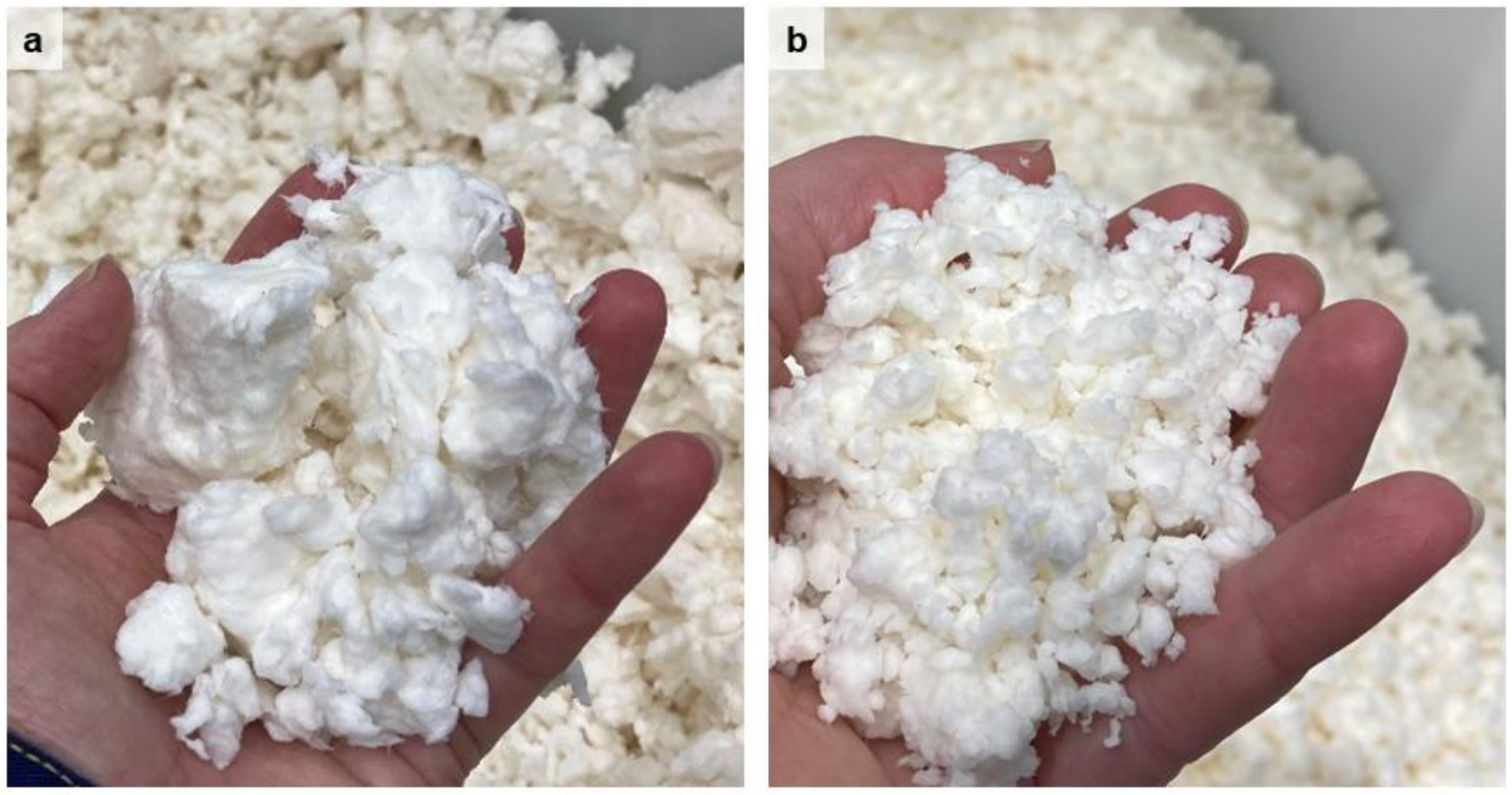

Figure S1. Screw-pressed pulps before foaming: a) SW and b) HW.

Table S1. Measured fiber properties of the SW and HW pulps.

Pulp type	Fiber length, mm	Fiber width, µm	Fines content, %	Coarseness, µm/m
SW	1.99 ± 0.01	28.3 ± 0.01	15.9 ± 0.07	150 ± 1.63
HW	0.76 ± 0.01	18.1 ± 0.07	18.7 ± 0.07	43.7 ± 0.57

Table S2. The used additives, abbreviations, dosages, and other information on the additives.

Additive	Abbreviation	Solution, %	Addition, % per dry fiber	Information	Provider	Curing
Guar gum	GG	Powder, dissolved in 1.2% solution	0.5	Fiber deflocculant, foam rheology modifier and dry-strength	Sigma-Aldrich	No
Sodium carboxymethyl cellulose	CMC	Powder, dissolved in 1% solution	0.5	Dry strength, foam rheology modifier, FF-4000G, medium Mw, medium DS (0.6-0.9)	Nouryon	No
Citric acid	CA	Powder, dissolved in 10% solution	1	Dry and wet strength	Sigma-Aldrich	190°, 10 min

Basic properties of pilot samples

Table S3. Target consistency, measured consistency and air content of the foams produced at pilot scale as well as the measured average grammage, thickness, density and pore size of the dried pilot samples. Grammage and thickness are average of 10 sheets having a size of ca. 15x12 cm. The average pore size was calculated from tomography images, with one 5x5 mm sample analyzed for each trial point.

TP number	Target fiber consistency, %	Measured consistency %	Measured air content %	Grammage g/m^2	Thickness µm	Density kg/m^3	Average pore size, µm
1	10	9.5	85	138 ± 13.3	6258 ± 1135	22.4 ± 2.0	400
2	10	8.3	84	128 ± 19.8	5914 ± 1139	21.9 ± 1.1	313
3	10	10.2	83	201 ± 17.4	7360 ± 749	27.3 ± 0.6	332
4	10	9.4	82	192 ± 26.0	5874 ± 853	32.8 ± 2.7	237
5	10	8.1	84	138 ± 22.9	4522 ± 577	30.4 ± 3.5	336
6	10	8.9	84	141 ± 18.5	6436 ± 707	21.8 ± 1.1	344
7	12	13.1	78	285 ± 94.7	6908 ± 2194	41.2 ± 2.6	217
8	14	17.6	78	668 ± 202	9944 ± 2032	66.6 ± 11.7	183
9	10	9.3	81	164 ± 13.1	6062 ± 627	27.1 ± 1.7	303
10	10	13.1	84	149 ± 27.4	5956 ± 1541	25.4 ± 2.0	353
11	10	10.5	80	572 ± 123	13370 ± 2669	42.7 ± 1.7	241

Advanced material characterization

Table S4. Final characterization plan.

TP number	Compression	Z-strength	Absorption	Tomography	Backlight	SEM	FTIR	Rheology*
1	x	x		x	x			x
2	x	x		x	x			x
3	x	x	x	x	x	x	x	x
4	x			x				x
5	x			x				x
6	x	x	x	x		x		x
7	x			x				
8	x	x		x				
9	x	x	x	x		x	x	
10	x		x	x		x	x	
11	x	x	x	x		x	x	x

* measured from the foam produced in a laboratory scale

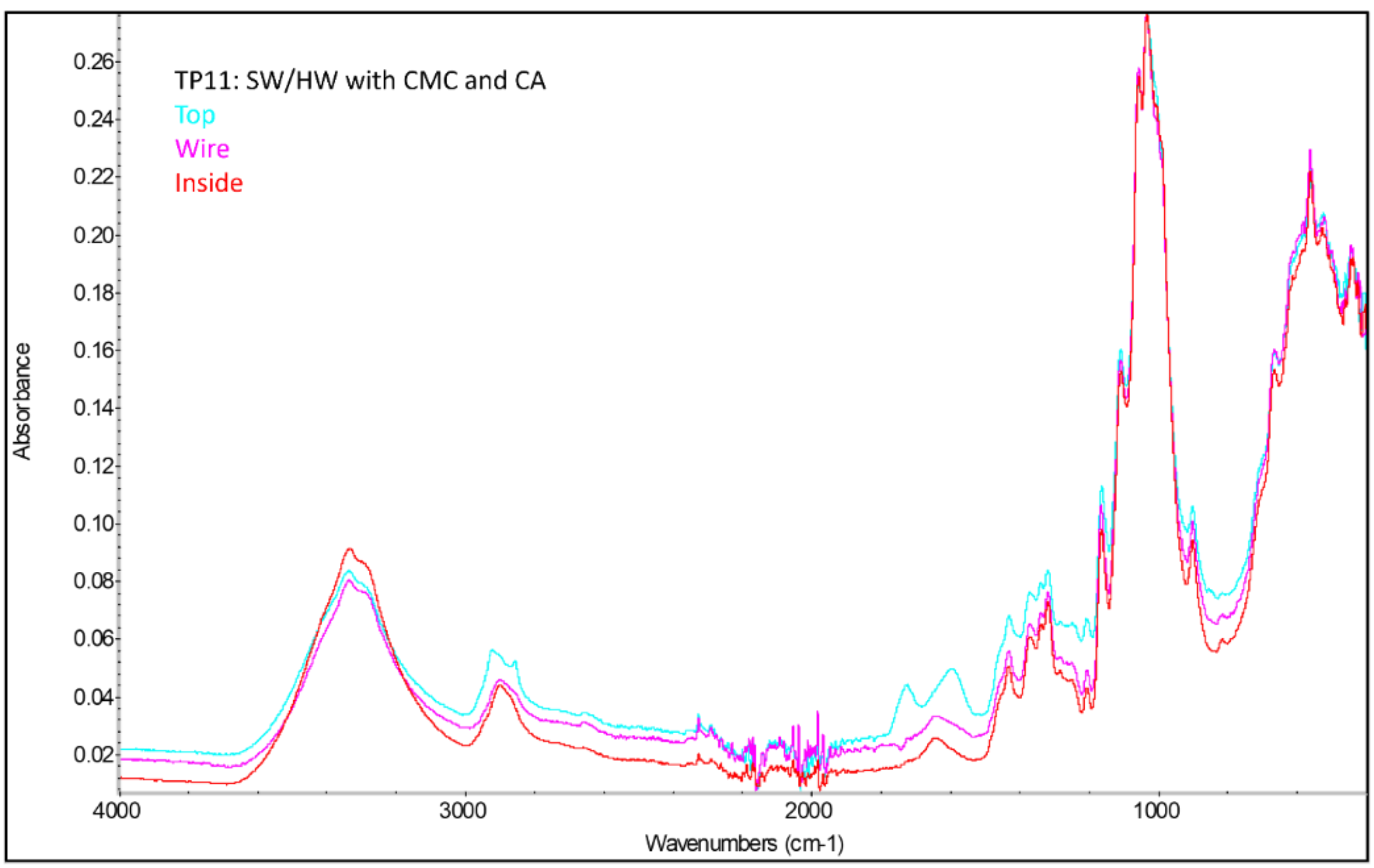

Figure S2. FTIR spectrum measured from the top, wire side, and middle of TP11 with CMC and CA.

Floc volume and size distribution from XμCT images

The flocs in the 3D XμCT images (Fig. S3) are determined as regions where the fibers are closer to each other than on average. To detect these regions, the following algorithm is used:

1. The original image is binarized so that fibers and background are separated. This image is called M_{fibers}.
2. The local thickness transform (LTT) of M_{fibers} is used to define the distances between the fibers. The result of LTT is a 3D image where each void space voxel has a value describing the diameter of the largest sphere that covers that voxel without overlapping with surrounding fibers. This image is called LT_{pores}. The mean value of LT_{pores} from the voxels belonging to the void space defines the mean fiber-fiber distance (D_{fiber}).
3. LTT is applied to the inverse of M_{fibers} to define the fiber thickness. This image is called LT_{fibers}.The mean value of LT_{fibers} from the voxels belonging to the fiber space defines the mean fiber thickness (T_{fiber}).
4. To find network regions where fibers are closer to each other than a threshold distance D, the following is done:
    a. LT_{pores} is thresholded using D as a threshold value. This gives us a mask of regions where the fiber-fiber distance is smaller than D. This image is called M_D.
    b. We add M_D to M_{fibers} so that the resulting image is a mask where all the fibers and the distances smaller than D are combined. This image is called M_{floc}.
    c. To remove the fibers not belonging to the floc area, a binary opening is made for M_{floc}. The opening is made with a kernel size of $2 \times T_{fiber} + D$. In essence, this removes the regions that are smaller than 2 fiber thicknesses from the image. The resulting image contains regions that are considered as flocs.
    d. To plot the floc volume and mean diameter, one finds all non-connected areas in the M_{floc}. Their volumes are given by the number of corresponding voxels, and diameters as those of spheres with corresponding volume. This data can be used to plot the total floc volume as a function of D or normalized D that is obtained by dividing D with D_{fiber}.
5. The mean floc diameter and relative total floc volume were determined within the selected normalized fiber-fiber distance range (0.89–1.87) across all trial points, aiming to obtain comparable average values while minimizing the influence of extreme outliers.

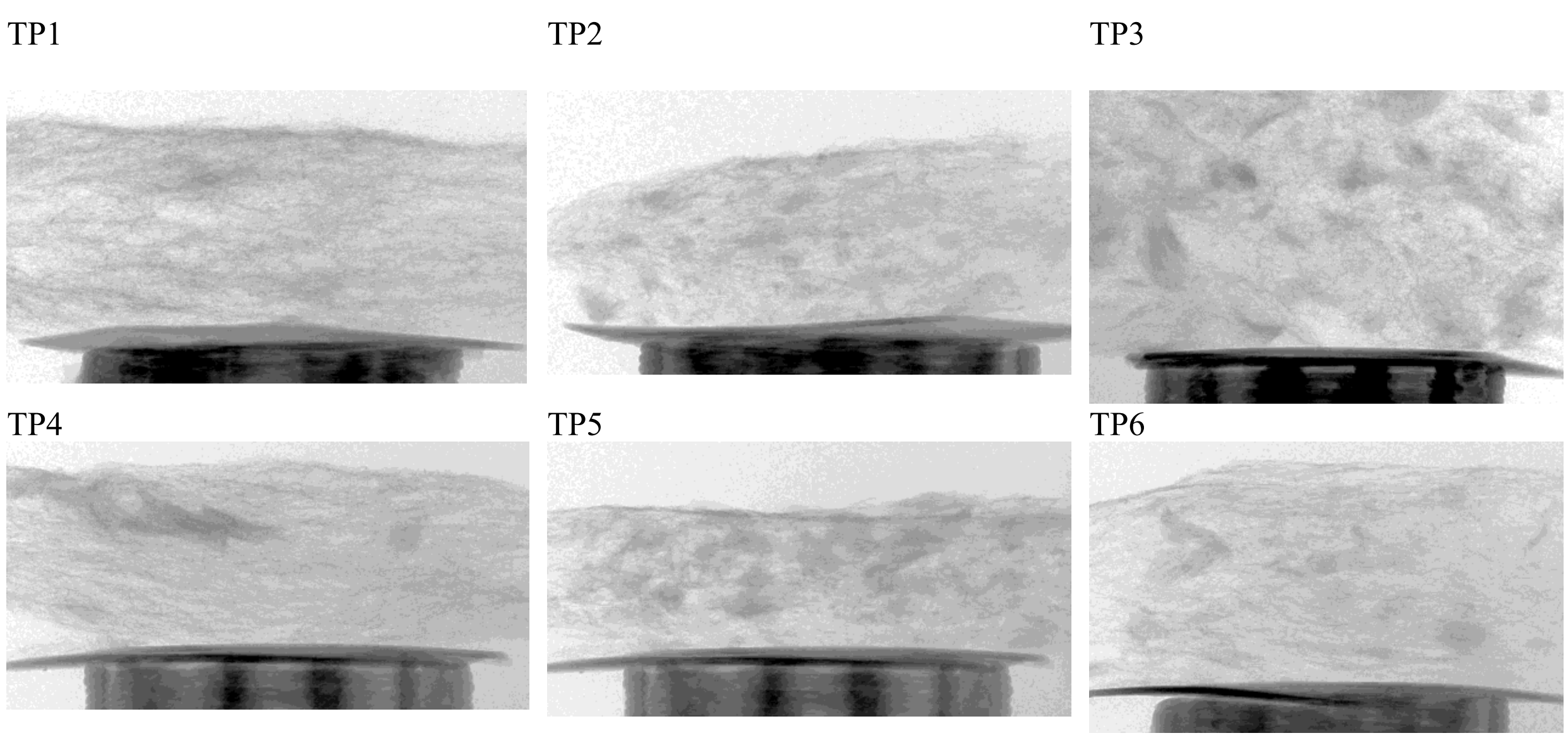

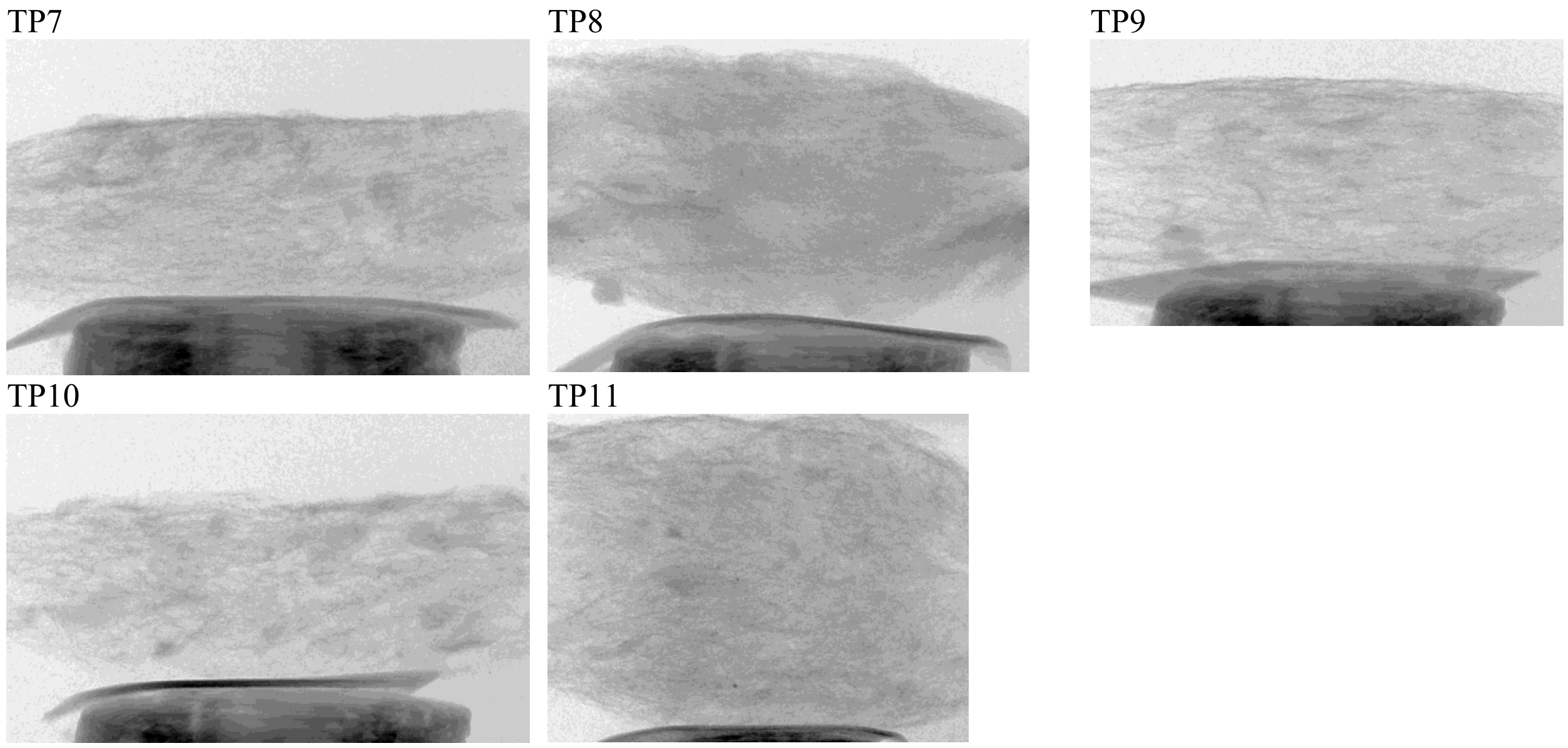

Figure S3. X-ray tomography images visualized using Voreen software. Contrast of the tomography images was adjusted to visualize the flocs better. No other modifications were applied.

Water absorption

Water absorption of the samples was measured to further evaluate if cross-linking between CA, CMC, and fibers has happened. Water absorption of the samples was measured using a modified ISO 12625-8 (Basket test). For the absorption measurement, a sample was cut into a size of 35mm x 70mm and placed in a designated basket. The basket was immersed in deionized water for 30 seconds, lifted out and allowed to drain for 60 seconds at a ca. 30-degree angle. Absorption of the sample was the wet sample weight divided by the dry sample weight. The results are presented as an average of two parallel measurements for the weak samples (TP3, TP9), and four parallel measurements for the samples (TP6, TP10, TP11) presenting higher wet strength. The dry weight of the samples was determined by drying at 105°C for 12 hours.

From the tested samples, the ones including CA had the highest water holding capacity. However, the measurement procedure was somewhat unreliable since most of the samples started to disintegrate. The sample including CMC+CA kept its shape best and did not collapse during testing, which can indicate strong bonds in the structure. These results indicate that even quite small amounts of CA can be used to improve the water absorption capacity of low-density materials, and better wet strength/shape-stability was achieved with the combination of CMC and CA.

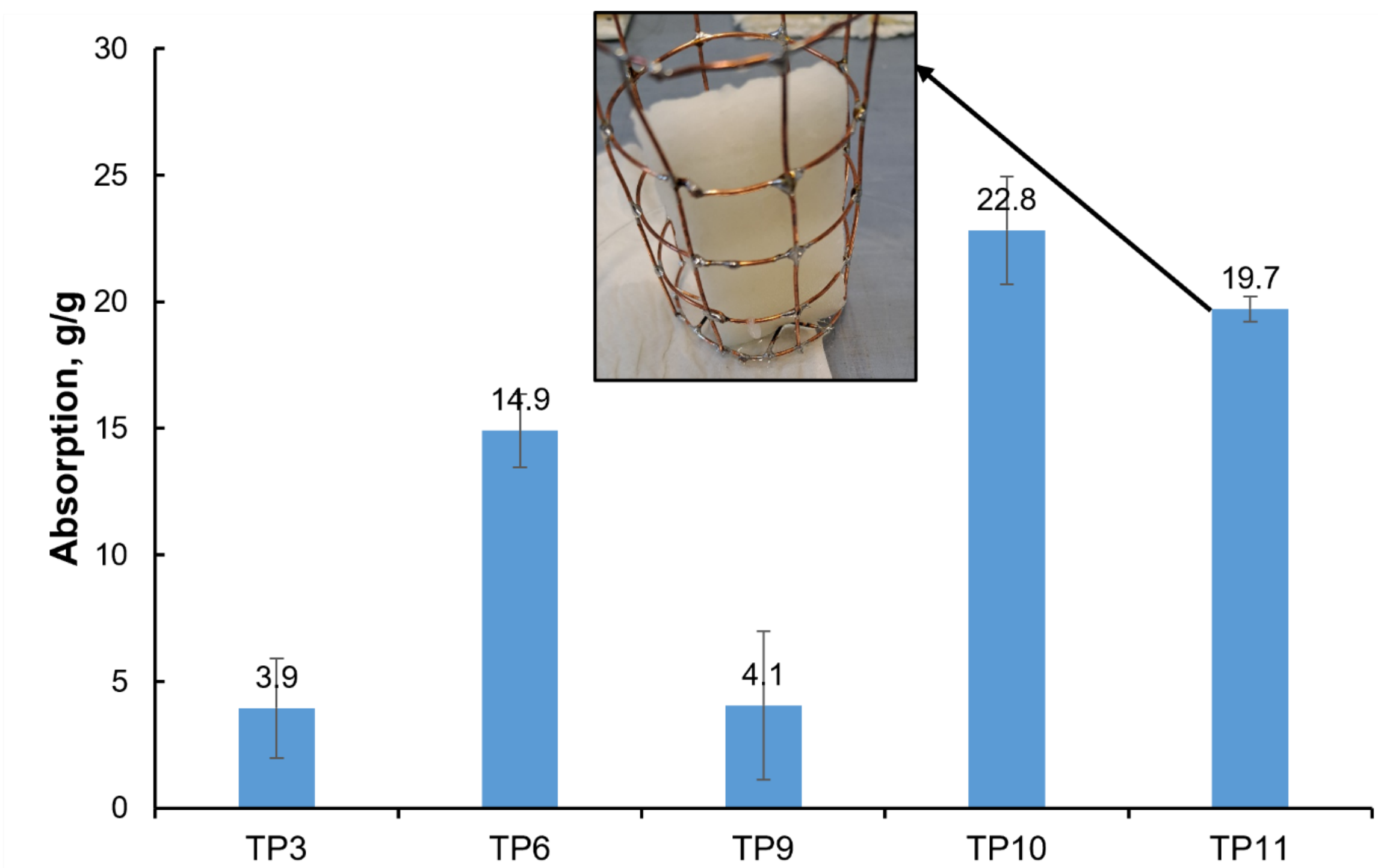

Figure S4. Water absorption of the samples was measured using a modified ISO 12625-8 (Basket test). The basket was immersed in deionized water for 30 seconds, lifted out and allowed to drain for 60 seconds at a ca. 30-degree angle. Absorption of the sample was the wet sample weight divided by the dry sample weight.

Mechanical tests

Table S5. Z-strength and the density of the sample that was used for the measurement.

TP	Formulation	Density kg/m^3	Z-strength kPa	StDev kPa	95% confidence interval kPa
TP1	SW	25.0	0.50	0.072	0.089
TP2	HW	23.6	0.16	0.029	0.036
TP3	SW/HW	29.8	0.53	0.099	0.122
TP6	SW/HW+GG 0.5%	23.5	1.5	0.141	0.176
TP8	SW/HW+GG 0.5%, 14%	68	5	1.093	1.357
TP9	SW/HW+CMC 0.5%	29	2.7	0.179	0.222
TP11	SW/HW+CMC 0.5% + CA 1%	44	12	0.954	1.185

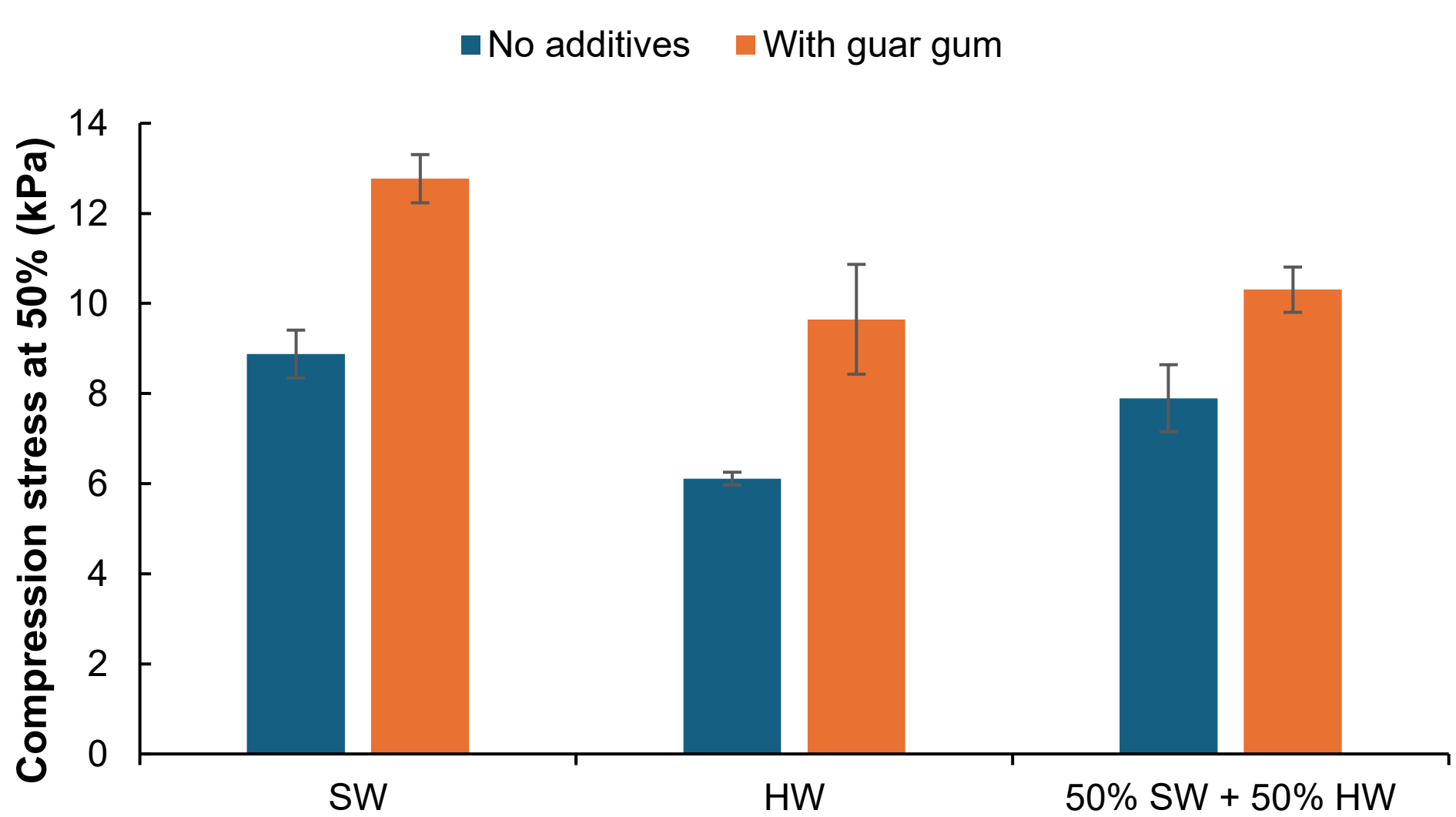

Figure S5. The density normalized compression stress results for SW, HW, and 50/50 SW/HW with and without GG.

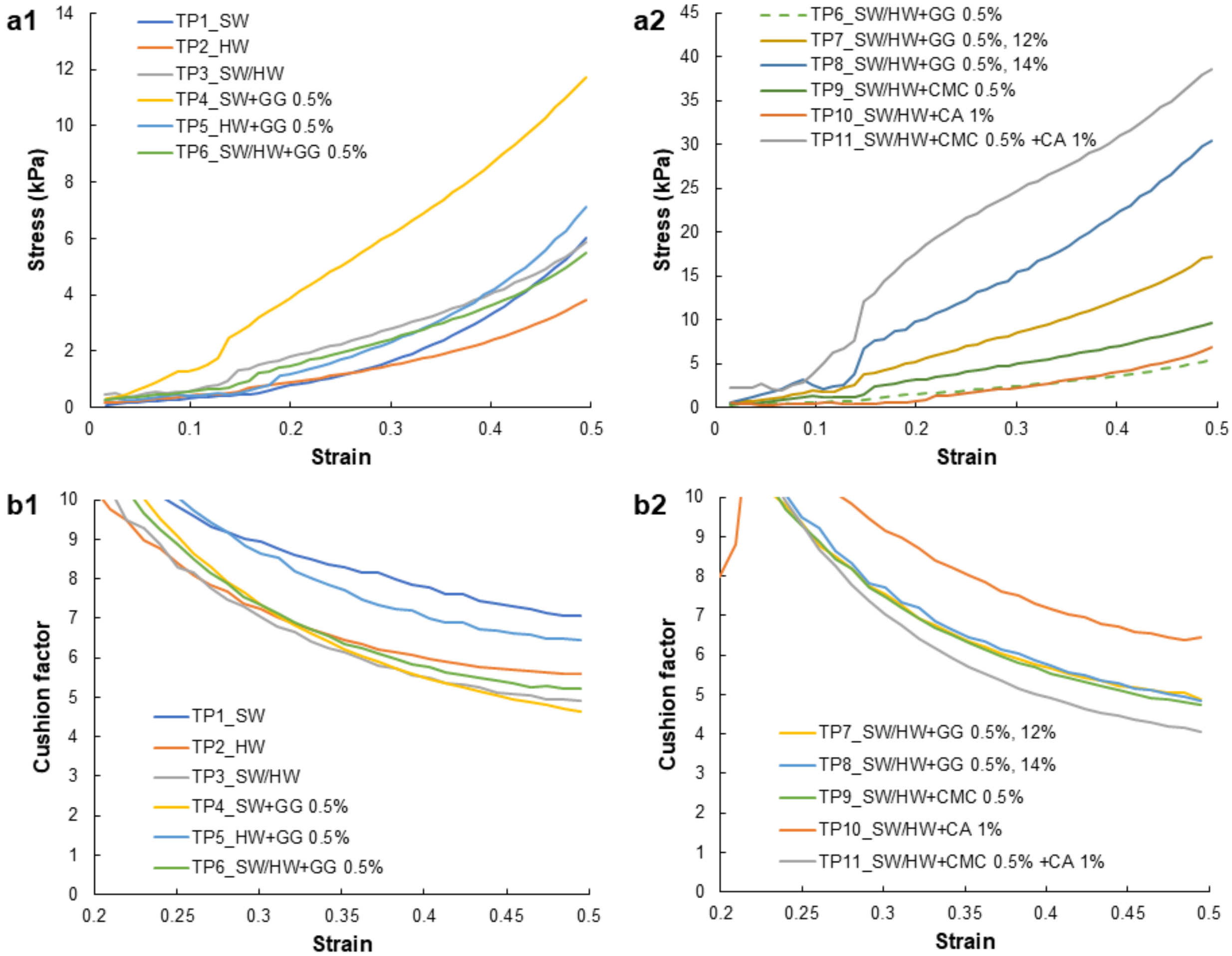

Figure S6. Stress-strain curves of (a1) TP1-TP6, and (a2) TP6-TP11, and cushion factor curves of (b1) TP1-TP6, and (b2) TP7-TP11.

Water-usage comparison for 1% and 14% consistency

The amount of water needed for the fiber suspensions in 1% and 14% consistencies was calculated as follows. Let the mass of fully dried product material be m_{ds}. The consistency Con can be written in terms of the total mass of water and solids, m_{total}, as

$$Con = \frac{m_{ds}}{m_{total}}. \quad \text{(S1)}$$

The corresponding total mass of water needed in the forming process is

$$m_{water} = m_{total} - m_{ds} = \frac{m_{ds}}{Con} - m_{ds}. \quad \text{(S2)}$$

These equations allow calculations of the water masses as presented in Table S6. For example, at a consistency of 1%, 1000 kg of dry solids corresponds to a total suspension mass of 100 000 kg, resulting in 99 000 kg of water. When processes are compared at 1% and 14% consistency, the water amounts needed for one ton of product are 99 m^3/t and 6.1 m^3/t, respectively.

Table S6. Comparison of the water amounts needed to produce 1000 kg of dry product material when suspension consistency is either 1% or 14%.

**Fiber consistency**	**Water needed (per kg/t abs. dry product)**	**Water (m^3/t)**
1%	99 000 kg	99 m^3
14%	6 140 kg	6.1 m^3
